# Suppression of T Cell Autophagy Results in Decreased Viability and Function of T Cells Through Accelerated Apoptosis in a Murine Sepsis Model*

**DOI:** 10.1097/CCM.0000000000002016

**Published:** 2016-12-16

**Authors:** Takehiko Oami, Eizo Watanabe, Masahiko Hatano, Satoshi Sunahara, Lisa Fujimura, Akemi Sakamoto, Chizuru Ito, Kiyotaka Toshimori, Shigeto Oda

**Affiliations:** 1 Department of Emergency and Critical Care Medicine, Chiba University Graduate School of Medicine, Chiba, Japan.; 2 Department of Biomedical Science, Graduate School of Medicine, Chiba University, Chiba, Japan.; 3 Department of Reproductive Biology and Medicine, Chiba University Graduate School of Medicine, Chiba, Japan.

**Keywords:** cecal ligation and puncture, cell death, critical care, immunosuppression

## Abstract

**Objective:**

While type 1 programmed cell death (apoptosis) of T cells leads to immunosuppression in sepsis, a crosstalk between apoptosis and autophagy (type 2 programmed cell death) has not been shown. The aim of this study is to elucidate the details of the interaction between autophagy and immunosuppression.

**Design:**

Laboratory investigation in the murine sepsis model.

**Setting:**

University laboratory.

**Subjects:**

Six- to 8-week-old male mice.

**Interventions:**

We investigated the kinetics of autophagy in T cells from spleen in a cecal ligation and puncture model with green fluorescent protein-microtubule-associated protein light chain 3 transgenic mice. We analyzed apoptosis, mitochondrial homeostasis and cytokine production in T cells, and survival rate after cecal ligation and puncture using T cell–specific autophagy-deficient mice.

**Measurements and Main Results:**

We observed an increase of autophagosomes, which was assessed by flow cytometry. However, an autophagy process in CD4^+^ T cells during sepsis was insufficient including the accumulation of p62. On the other hand, a blockade of autophagy accelerated T cell apoptosis compared with the control mice, augmenting the gene expression of Bcl-2-like 11 and programmed cell death 1. Furthermore, mitochondrial accumulation in T cells occurred via a blockade of autophagy during sepsis. In addition, interleukin-10 production in CD4^+^ T cells from the cecal ligation and puncture–operated knockout mice was markedly increased. Consequently, deficiency of autophagy in T cells significantly decreased the survival rate in the murine sepsis model.

**Conclusions:**

We demonstrated that blocking autophagy accelerated apoptosis and increased mortality in concordance with the insufficient autophagy process in CD4^+^ T cells in the murine sepsis model, suggesting that T cell autophagy plays a protective role against apoptosis and immunosuppression in sepsis.

## Abstract 2

Supplemental Digital Content is available in the text.

Sepsis remains a leading cause of death in the world, regardless of advances in the critical care field (1). It has also been reported that a substantial number of sepsis patients surviving the initial hyperinflammatory phase of the disorder have a poor long-term outcome and low quality of life (2). Recently, a new disease concept, persistent inflammation, immunosuppression, and catabolism syndrome (PICS), was proposed to assess immunosuppression and nutritional status for patients with prolonged ICU length of stay (3). Additionally, it can contribute to mortality in the late phase of sepsis (4).

Immunosuppression in sepsis is mainly caused by apoptosis of several immune cell types including macrophages, T cells, B cells, and dendritic cells. In particular, CD4^+^ T cells are reported to have considerable involvement in the pathophysiology of immunosuppression (5, 6). Several treatments to prevent immunosuppression have targeted apoptosis of immune cells in animal models (7–10). However, no definitive treatments for immunosuppression in clinical practice have been established so far, and effective treatments are still required to improve the late phase mortality of sepsis.

Apoptosis is known as “type 1 programmed cell death,” and autophagy as “type 2 programmed cell death.” Although a crosstalk between both types of cell death exists, the details of pathophysiology in sepsis have not been elucidated (11). Autophagy is a protein degradation system that is essential for cellular homeostasis. Its main functions are to recycle proteins, remove damaged organelles, eliminate microorganisms, and play a role in antigen presentation. The process of autophagy begins with the formation of an isolation membrane, which elongates to eventually form a double-membrane vesicle. Several autophagy genes, Atg3, Atg5, and Atg7, and microtubule-associated protein light chain 3 (LC3) are involved in the process (12). Recent clinical investigations and animal experiments have demonstrated that autophagy plays a protective role in several organs during sepsis (13–15). However, so far the relationship between immunosuppression and autophagy in sepsis has not been well documented. A recent study has shown that a deficiency in T cell autophagy increases mortality and immunosuppression using the cecal ligation and puncture (CLP) model (16). In this study, the T cell–specific Atg7 knockout was employed. However, the details of the mechanisms by which deficiency of autophagy induces apoptosis have not been demonstrated.

In our study, we used T cell–specific Atg5 knockout mice (CD4-Cre recombinase/Atg5^f/f^ mice: CD4-Cre/Atg5^f/f^ mice) and performed a CLP procedure to prove our hypothesis that autophagy is related to apoptosis in sepsis. Consequently, we demonstrated the kinetics of autophagy in T cells during sepsis, crosstalk between autophagy and apoptosis, mitochondrial accumulation by deficiency of autophagy, and augmented interleukin (IL)-10 production by CD4^+^ T cells lacking autophagy. Based on the above results, we will discuss the influence on apoptosis and immunosuppression by blocking T cell autophagy in sepsis.

## MATERIALS AND METHODS

### Mice

Six- to 8-week-old male mice (C57BL/6) were used in the animal experiments. We used only male mice to minimize a variability of experimental results due to gender differences. Green fluorescent protein (GFP)-LC3 transgenic mice and Atg5^f/f^ mice were kindly gifted. Transgenic Atg5^f/f^ mice and CD4-Cre recombinase mice were crossed to generate T cell–specific Atg5 knockout mice (CD4-Cre/Atg5^f/f^ mice), which deleted Atg5 gene in T cell by Cre-recombinase expression. All mice were acclimated to a 12-hour day/night cycle under specific pathogen-free conditions with food at least 1 week before the experiments. All experimental procedures were performed in strict accordance with the National Institute of Health guidelines and were approved by the Institutional Animal Care and Use Committees of Chiba University.

### CLP

The above procedures were performed on mice as previously described (13). The details of the procedure are described in the **supplementary data** (Supplemental Digital Content 1, http://links.lww.com/CCM/C114). Sham model mice were operated same as the CLP model, except for cecum ligation and puncture. Mice were euthanized at 6 and 24 hours to collect samples. Survival was observed every 12 hours and killed when they were moribund.

### Lymphocytes Isolation and Flow Cytometry Analysis

Spleens were removed from anesthetized mice surgically and pressed with slide glasses gently. Then, they were washed with phosphate buffered saline, and RBCs were lysed with ammonium-chloride-potassium lysis buffer. Peripheral blood was obtained from inferior vena cava, and RBCs were lysed with distilled water. Centrifuged lymphocytes were resuspended in RPMI-1640 medium (Sigma-Aldrich, St. Louis, MO).

Cell number and viability of lymphocytes resuspended in RPMI-1640 medium were quantified with TC20 Automated cell counter (Bio Rad, Hercules, CA).

Surface marker staining, intracellular staining of p62, apoptosis, mitochondrial staining, and lysosomal staining were performed and analyzed with FACS caliber (BD Bioscience, San Jose, CA). Antibodies and reagents used in these experiments and detailed methods are described in the supplementary data (Supplemental Digital Content 1, http://links.lww.com/CCM/C114).

### Cell Sorting

Prepared splenocytes were stained with anti-CD4 biotin antibody on ice for 30 minutes and then incubated with magnetic-streptavidin (Miltenyi Biotec, Bergisch Gladbach, Germany) for 15 minutes after washing. After resuspended in cell sorting buffer, CD4^+^ T cells were isolated with separate columns (Miltenyi Biotec) by negative selection. The purity of the CD4^+^ T cells was determined for more than 90%. The sorted cells were used for Western blotting, real time quantitative reverse transcription polymerase chain reaction, electron microscopic analysis, and cytokine secretion analysis. Detailed methods are described in the supplementary data (Supplemental Digital Content 1, http://links.lww.com/CCM/C114).

### Statistical Analysis

Data are presented as mean ± sd or absolute numbers and percentages as appropriate. We tested for differences between the two groups using an unpaired *t* test for continuous data and used two-way analysis of variance among different categoric independent variables. Statistical analyzes were conducted using the GraphPad Prism 6 (GraphPad Software, San Diego, CA).

## RESULTS

**Although Lymphocyte Autophagosomes are Increased by Septic Stimulation, the Process of Autophagy is Insufficient in a Murine Sepsis Model in CD4+ T Cells**

**Figure 1.**
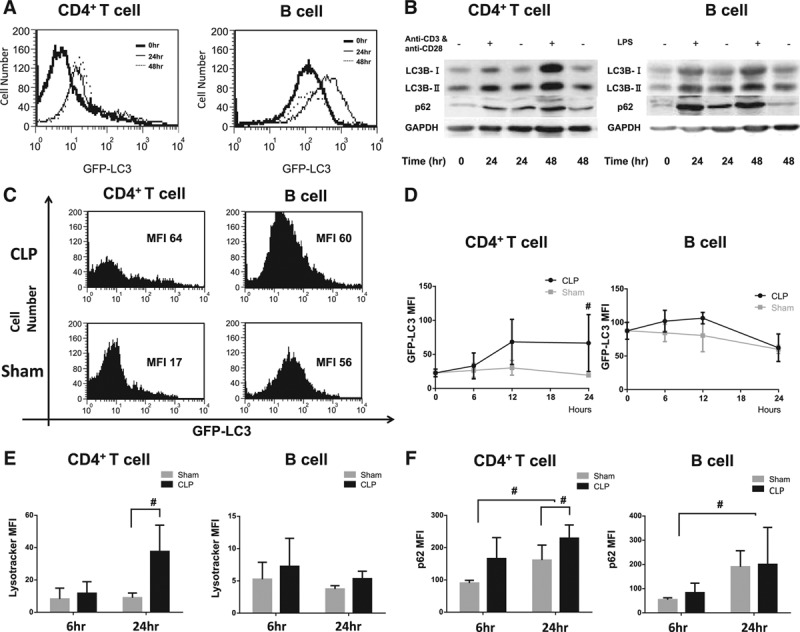
Although lymphocyte autophagosomes are increased by septic stimulation, the process of autophagy is insufficient in a murine sepsis model in CD4^+^ T cells. **A**, Evaluation of autophagy in green fluorescent protein (GFP)-light chain 3 (LC3) transgenic mice by flow cytometry. After incubation with anti-CD3 and anti-CD28 antibodies for 48 hr for CD4^+^ lymphocytes and lipopolysaccharide (LPS) for B lymphocytes, mean fluorescence intensity (MFI) of harvested splenocytes was measured by flow cytometry for 48 hr. Representative data of three independent experiments are shown. **B**, Quantitation of autophagic protein, LC3-B and p62, was performed by western blotting. Lymphocytes were stimulated as the above procedure. The amount of each protein level was normalized by GAPDH. Representative data of independent three experiments are shown. **C**, FACS profiles for harvested splenocytes of GFP-LC3 transgenic mice. Mice underwent cecal ligation and puncture (CLP) and sham procedure and were killed at the time of 24 hr after the operation. Harvested splenocytes were stained with surface antigen markers and measured by flow cytometry. Representative data of five independent experiments are shown. **D**, Sequential MFI of lymphocytes from GFP-LC3 transgenic mice was compared between CLP- and sham-operated mice. *n* = 3–6 mice in each group. Results are shown as mean ± sd in a bar graph. Data are analyzed by student *t* test; #*p* < 0.05. **E**, MFI of Lysotracker staining lymphocytes from sham- and CLP-operated mice are shown. Each sample was stained with surface antigen markers. Data are expressed as mean and sd, and analyzed by two-way analysis of variance (ANOVA) and student *t* test. *n* = 3–4 mice in each group; #*p* < 0.05. **F**, MFI of p62 protein conjugated with fluorescent second antibody in lymphocytes from wild-type mice. Mice underwent CLP and sham procedure, and were killed at the time of 24 hr after the operation. Harvested splenocytes were stained with p62 and surface antigen markers concomitantly, and then measured by flow cytometry. Data are expressed as mean and sd, and analyzed by two-way ANOVA and student *t* test. *n* = 4–6 mice in each group; #*p* < 0.05. FACS = fluorescence activated cell sorting, GAPDH = glyceraldehyde-3-phosphate dehydrogenase.

To evaluate the autophagy kinetics of lymphocytes in sepsis, we performed in vitro assay to replicate the condition at first. Lymphocytes from GFP-LC3 mice were stimulated with anti-CD3/CD28 or lipopolysaccharide (LPS) for 48 hours. Mean fluorescence intensity (MFI) of GFP-LC3, which represents autophagosomes, was measured by flow cytometry. As shown in **Figure 1*A***, MFI of GFP-LC3 in CD4^+^ T cells increased after activation. In B cells, the intensity transiently increased at 24 hours after the activation but declined by 48 hours. LC3B-II levels in CD4^+^ T cells and B cells were also increased by the stimulation sequentially in the Western blotting analysis. To determine the condition of autophagy flux, further analyses regarding selective substrates were conducted. P62, a marker protein for insufficient/inhibition of autophagy flux, was also increased in CD4^+^ T cells and B cells while LC3B-II levels were increased (**Fig. 1*B***). These results suggest that autophagy in immune cells works insufficiently by mimicking septic stimulation despite increased appearance of autophagosomes.

Based on the above results, we performed in vivo assay using a murine sepsis model. A CLP procedure was performed on GFP-LC3 mice and measured MFI of GFP-LC3 by flow cytometry. Autophagosomes, which were assessed by the MFI of GFP-LC3, were significantly increased in the CLP model over time, in CD4^+^ T cells (**Fig. 1, *C*** and ***D***). However, no difference was observed in B cells between CLP- and sham-operated mice. Then, we investigated the lysosome kinetics with Lysotracker Red DND-99 by flow cytometry. Same as the kinetics of autophagosomes, MFI of Lysotracker in CD4^+^ T cells but not B cells was increased in the CLP-operated mice 24 hours after the procedure (**Fig. 1*E***). As autophagosomes are subject to fusion with lysosomes to accomplish the autophagy process, these results indicated lysosomes accumulated in CD4^+^ T cells. To investigate the autophagy flux in the sepsis model, p62 protein levels of lymphocytes were measured by flow cytometry. MFI of p62 in the CLP mice was increased in CD4^+^ T cells but not in B cells after 24 hours compared with the sham-operated mice (**Fig. 1*F***). We confirmed a double-membrane bound structure with membrane/organellar debris within and heterolysosomes/autolysosomes in CD4^+^ T cells from CLP-operated mice with transmission electron microscope (TEM) (**Supplemental** Fig. 1, Supplemental Digital Content 2, http://links.lww.com/CCM/C115; legend, Supplemental Digital Content 1, http://links.lww.com/CCM/C114). Although it is suggested autophagosomes are increased by septic stimulation, autophagy process is insufficient for the CLP-operated mice.

### Apoptosis Was Accelerated by Atg5 Deletion in T Cells During Sepsis

**Figure 2.**
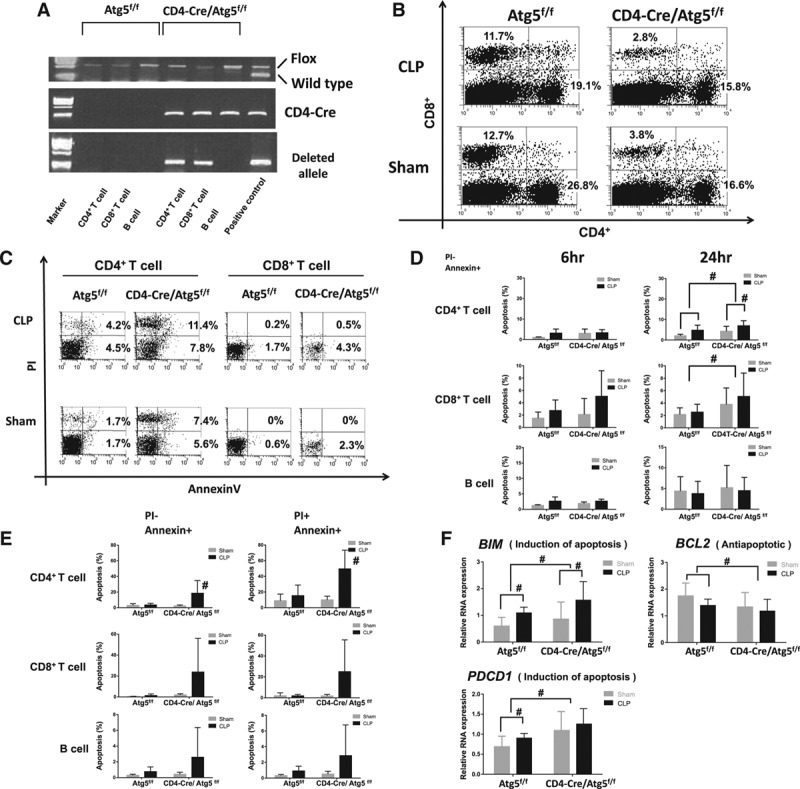
Apoptosis was accelerated by Atg5 deletion in T cells during sepsis. **A**, Polymerase chain reaction amplification of genomic DNA from sorted lymphocytes in Atg5^f/f^ and CD4-Cre/Atg5^f/f^ mice. Atg5 flox allele and CD4-Cre allele are shown in the upper and middle figure, respectively. The positive controls are tail DNA from CD4-Cre/Atg5^f/f^ mice. In the lower figure, the deleted allele is found in CD4^+^ T cells and CD8^+^ T cells extracted from CD4-Cre/Atg5^f/f^ mice. The positive control is liver DNA from liver specific-Cre/Atg5^f/f^ mice. **B**, Representative subpopulation of splenocytes in Atg5^f/f^ and CD4-Cre/Atg5^f/f^ mice. Mice were performed cecal ligation and puncture (CLP) or sham procedure. Splenocytes were stained with anti-CD4^+^/PE and anti-CD8^+^/APC. Representative data of five independent experiments are shown. **C**, FACS profiles of splenocytes stained with Annexin V and PI in Atg5^f/f^ and CD4-Cre/Atg5^f/f^ mice for 24 hr postoperatively. Samples were stained with anti-CD4^+^/PE and anti-CD8^+^/APC. Representative data of five independent experiments are shown. **D**, Subpopulation of Annexin V positive and PI negative staining splenocytes were shown for early (postoperative period, 6 hr) and late (postoperative period, 24 hr) apoptosis. Data are expressed as mean and sd; *n* = 8–10 mice in each group; #*p* < 0.05 was significance analyzed by two-way analysis of variance (ANOVA) and student *t* test. **E**, Subpopulation of Annexin V and PI staining lymphocytes from peripheral blood were shown for early (Annexin V positive and PI negative) and late (Annexin V positive and PI positive) phase of apoptosis. Data are expressed as mean and sd; *n* = 6–8 mice in each group; #*p* < 0.05 was significance analyzed by student *t* test between CLP-operated Atg5^f/f^ mice and CLP-operated CD4-Cre/Atg5^f/f^ mice. **F**, Relative RNA expression for apoptotic gene in sham, CLP-operated Atg5^f/f^ mice and sham, and CLP-operated CD4-Cre/Atg5^f/f^ mice. Total RNA in CD4^+^ splenic lymphocytes was extracted from experimental mice at 24 hr after the procedure, and then relative RNA expression for the several genes was analyzed. Data are expressed as mean and sd; *n* = 8–10 mice in each group; #*p* < 0.05 was significance analyzed by two-way ANOVA and student *t* test. APC = allophycocyanin, Bcl-2 = B-cell leukemia/lymphoma 2, BIM = Bcl-2-like 11, FACS = fluorescence activated cell sorting, PDCD1 = programmed cell death 1, PE = phycoerythrin, PI = propidium iodide.

To investigate the role of autophagy in sepsis, we generated T cell–specific autophagy knockout mice (CD4-Cre/Atg5^f/f^) and performed the CLP procedure. We confirmed the deleted allele in CD4^+^ T cells and CD8^+^ T cells obtained from CD4-Cre/Atg5^f/f^ mice (**Fig. 2*A***). The CLP procedure caused decrease of CD4^+^ and CD8^+^ T cells from spleen in the control mice. In CD4-Cre/Atg5^f/f^ mice, percentages of CD4^+^ and CD8^+^ T cells from spleen decreased even in the sham-operated group (**Fig. 2*B***). Since CD4-Cre/Atg5^f/f^ mice are susceptible to the sham operation, we examined the levels of apoptosis in the autophagy-deficient mice in T cells. Annexin V positive CD4^+^ and CD8^+^ splenocytes were increased in CD4-Cre/Atg5^f/f^ mice 24 hours after the operation (Fig. 2*B*). Significant differences in the proportions of apoptotic CD4^+^ T cells and CD8^+^ T cells were found between CD4-Cre/Atg5^f/f^ mice and the control mice, and also between CLP- and sham-operated mice in CD4-Cre/Atg5^f/f^ group (**Fig. 2*D***). On the contrary, we did not observe any difference in B cells. Furthermore, we found significant differences in the proportions of apoptotic CD4^+^ T cells and CD8^+^ T cells from peripheral blood between CLP-operated Atg5^f/f^ mice and CLP-operated CD4-Cre/Atg5^f/f^ mice (**Fig. 2*E***).

The expression of Bcl-2-like 11 (BIM) and programmed cell death 1 (PDCD1), which have roles of apoptosis induction, was increased in CD4^+^ T cells from CD4-Cre/Atg5^f/f^ mice. Otherwise, the gene expression of B-cell leukemia/lymphoma 2, which hampers apoptosis induction, was decreased in CD4^+^ T cells from CD4-Cre/Atg5^f/f^ mice. Additionally, significant differences between CLP- and sham-operated CD4-Cre/Atg5^f/f^ mice were observed in the gene expression of BIM (**Fig. 2*F***). Based on the above results, it was suggested that deletion of T cell autophagy caused acceleration of apoptosis in the CLP model.

### Mitochondrial Mass Was Increased by Autophagy Deficiency in T Cells

**Figure 3.**
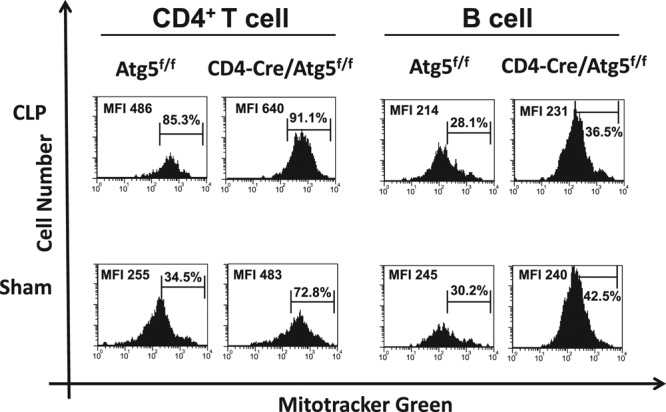
Mitochondrial mass was increased by autophagy deficiency in T cells. FACS profiles of Mitotracker FM green staining lymphocytes in sham, cecal ligation and puncture (CLP)-operated Atg5^f/f^ mice and sham, and CLP-operated CD4-Cre/Atg5^f/f^ mice at 24 hr after the procedure. Representative data of five independent experiments are shown. FACS = fluorescence activated cell sorting, MFI = mean fluorescence intensity.

As autophagy has a crucial role to remove damaged organelles including mitochondria, we speculated that blocking autophagy would affect mitochondrial function in T cells during sepsis. Mitochondrial mass, which was assessed by MFI of Mitotracker FM green, was significantly increased by the CLP procedure in the control mice (**Fig. 3**). Deficiency of T cell autophagy in CD4-Cre/Atg5^f/f^ mice increased mitochondrial mass by both of the sham and CLP operation (Fig. 3). However, no differences were found between the CLP model and sham model in B cells from CD4-Cre/Atg5^f/f^ (Fig. 3). As apoptosis was induced by autophagy deficiency in T cells from the above results, we evaluated mitochondrial membrane potential (MMP). In the control mice, relative MMP was increased after the CLP procedure. No increase of MMP was observed in autophagy-deficient CD4^+^ T cells. Furthermore, relative MMP in the CLP-operated Atg5 knockout mice is significantly lower than that of the control mice (**Supplemental** Fig. 2, Supplemental Digital Content 3, http://links.lww.com/CCM/C116; legend, Supplemental Digital Content 1, http://links.lww.com/CCM/C114).

### IL-10 Secretion Was Increased by Suppression of Autophagy in T Cells

**Figure 4.**
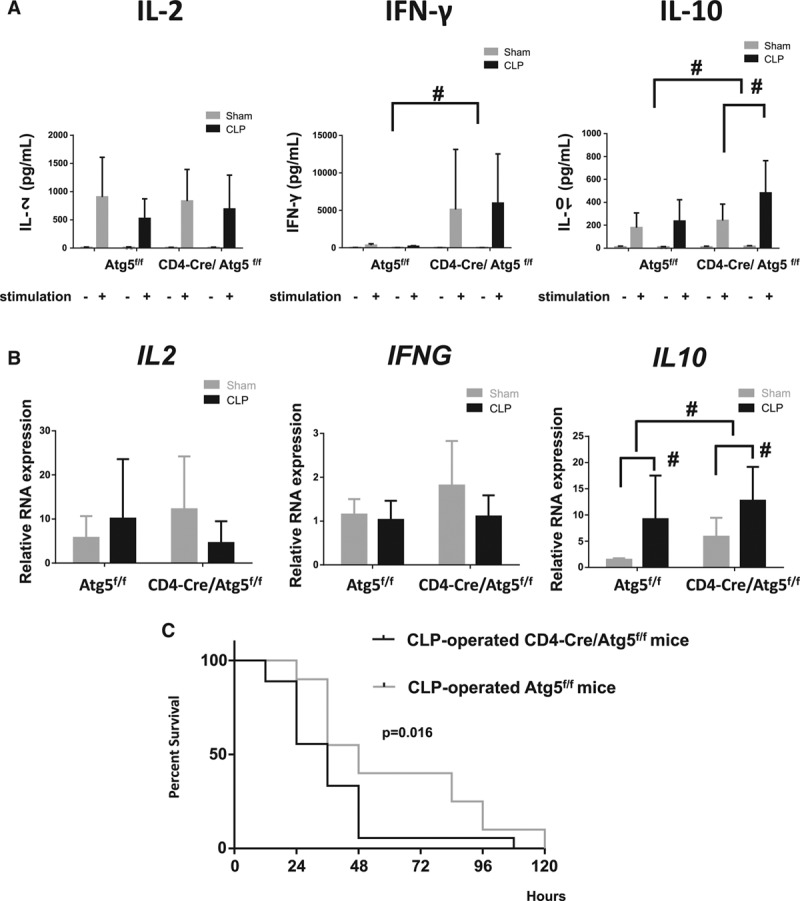
Interleukin (IL)-10 secretion was increased by blocking autophagy in T cells, and T cell autophagy affects the late phase mortality of sepsis. **A**, After lymphocytes were harvested from spleen, they were stimulated with anti-CD3 and anti-CD28 antibodies for 24 hr. Then, we measured IL-2, interferon (IFN)-γ, and IL-10 concentrations in the supernatant fluid of incubated lymphocytes by enzyme-linked immunosorbent assay. Data are expressed as mean and sd; *n* = 8–9 mice in each group; #*p* < 0.05 was significance analyzed by two-way analysis of variance (ANOVA) and student *t* test. **B**, Relative RNA expression for cytokine gene in sham, cecal ligation and puncture (CLP)-operated Atg5^f/f^ and sham, and CLP-operated CD4-Cre/Atg5^f/f^ mice. Total RNA in CD4^+^ lymphocytes was extracted from experimental mice at 24 hr after the procedure, and then relative RNA expression of IL-2, interferon-γ (IFNG) and IL-10 were analyzed. Data are expressed as mean and sd; *n* = 8–10 mice in each group; #*p* < 0.05 was significance analyzed by two-way ANOVA and student *t* test. **C**, Survival rates between CLP-operated Atg5^f/f^ mice and CLP-operated CD4-Cre/Atg5^f/f^ mice. Survival rates were observed every 12 hr. *n* = 18–20 mice in each group. *p* < 0.05 was significance analyzed by Kaplan-Meier method and log-rank test.

We investigated the cytokine secretion in the supernatant from stimulated CD4^+^ T cells. IL-10 concentrations in the CLP-operated CD4-Cre/Atg5^f/f^ mice were significantly increased (**Fig. 4*A***). This was also confirmed by messenger RNA expression (**Fig. 4*B***). Similarly, interferon (IFN)-γ and IL-4 concentrations were increased in CD4-Cre/Atg5^f/f^ mice. However, no difference was observed between the CLP- and sham-operated CD4-Cre/Atg5^f/f^ mice (Fig. 4*A*; and **Supplemental** Fig. 3, Supplemental Digital Content 4, http://links.lww.com/CCM/C117; legend, Supplemental Digital Content 1, http://links.lww.com/CCM/C114).

### Autophagy Deficiency in T Cells Decreases the Survival Rate in the Sepsis Murine Model

Because apoptotic cell death in T cells was the most prominent at 24 hours after CLP surgery (Fig. 2, *C–E*), we needed to evaluate the survival using a model that live longer than 24 hours at least. Therefore, we performed a survival study using a less severe CLP model. The mortality is supposed to be lowered than the previous model at the same time course. To verify this condition, we used a smaller needle (27G) for puncture instead of a 23G needle. Consequently, we found that the mortality rates in CD4-Cre/Atg5^f/f^ mice significantly increased compared with the control mice (*p* = 0.016) (**Fig. 4*C***). As shown in Figure 4*C*, the mortality rate for controls relative to the conditional knockout mice was a more robust improvement in survival at 72 hours, that is, just after occurrence of the T cell apoptosis.

## DISCUSSION

In this study, we demonstrated several findings regarding T cell autophagy in sepsis. First, an autophagy process in CD4^+^ T cells is insufficient during sepsis despite an increase of autophagosomes. Second, blockade of T cell autophagy accelerates apoptosis. Third, mitochondrial accumulation in T cells occurs via a blockade of autophagy during sepsis. Fourth, IL-10 production is increased in CD4^+^ T cells by blockade of autophagy, which may drive a further immunosuppressive state. Finally, a deficiency of autophagy in T cells decreases the survival rate in the murine sepsis model. These findings suggest that T cell autophagy plays a protective role against apoptosis and immunosuppression in the murine sepsis model.

As interest in the process of autophagy has developed, Crouser et al (17) have shed light on the interaction between autophagy and sepsis. In their report, they demonstrated that mitochondrial depletion was related to the removal of the damaged organelles by autophagy in a murine sepsis model. Furthermore, Watanabe et al (18) observed an increase of liver autophagosomes in sepsis patients for the first time. Although the role of autophagy in sepsis had not been clearly understood, Takahashi et al (13) demonstrated a protective role of autophagy in liver using CLP-operated mice. However, the kinetics of autophagy in T cells during sepsis have still not been elucidated. We used GFP-LC3 transgenic mice in which the autophagic activity can be monitored objectively over time using flow cytometry (19). Furthermore, we separated CD4^+^ T cells and confirmed the images of increased autophagosomes and autolysosomes in CLP relative to sham with our TEM data (Supplemental Fig. 1, Supplemental Digital Content 2, http://links.lww.com/CCM/C115; legend, Supplemental Digital Content 1, http://links.lww.com/CCM/C114). Also, we found remarkable mitochondrial damages in CD4^+^ T cells of CLP-operated mice. To our knowledge, ours is the only study in which T cell autophagy in sepsis was monitored in such detail to date.

Our results demonstrated that mitochondria accumulated in the T cells of the CD4-Cre/Atg5^f/f^ mice. It is consistent with the previous report that T cells lacking the autophagy-related genes Atg5 or Atg7 have inferior survival rates to those expressing the genes and contain expanded mitochondria (20, 21). As damaged organelles accumulate, cells are unable to maintain their physiologic functions. Although we did not demonstrate a direct relationship between apoptosis induction and mitochondrial accumulation, it has previously been shown that deletion of autophagy in T cells induces abnormal reactive oxygen species (ROS) production and apoptosis, and leads to increased mitochondrial content (21). From the above findings, it is suggested that autophagy plays a critical role to maintain mitochondrial homeostasis in sepsis.

**Figure 5.**
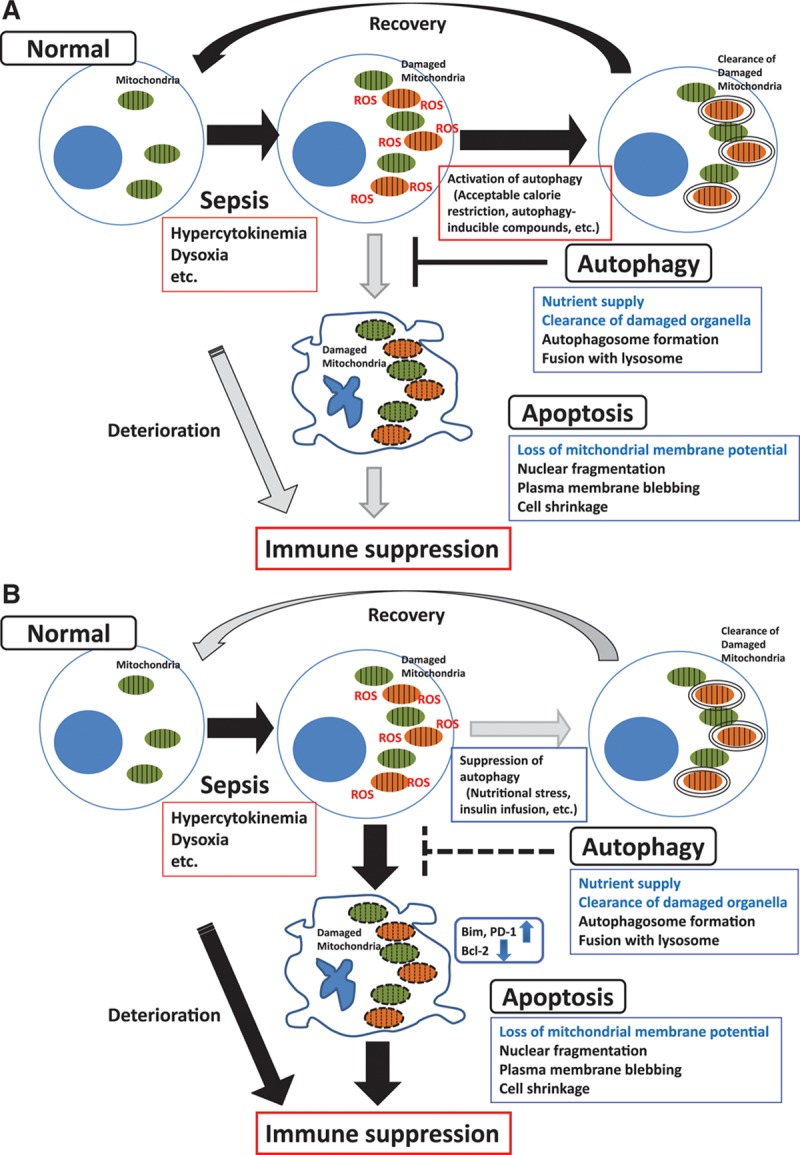
Interaction between autophagy and apoptosis in T cell. The summary figure illustrates the crosstalk between autophagy and apoptosis in T cells during sepsis. **A**, Septic stresses cause the increase of reactive oxygen species (ROS) and accumulation of damaged mitochondria. Damaged organelles are cleared by autophagic machinery and these events prevent apoptosis. **B**, Blocking T cell autophagy accelerates T cell apoptosis and eventually results in the immunosuppressive status. Bcl-2 = B-cell leukemia/lymphoma 2, PDCD1 = programmed cell death 1.

We demonstrated the intimate interaction between autophagy and apoptosis in T cells. Although sepsis causes the increase of ROS and accumulation of damaged mitochondria, damaged mitochondria is removed by autophagic machinery to prevent apoptosis (**Fig. 5*A***). On the other hand, blocking T cell autophagy causes accumulation of damaged mitochondria and acceleration of T cell apoptosis with augmented expression of BIM and PDCD1. Eventually, these changes result in the immunosuppressive status (Fig. 5*B*). It is supposed that sepsis patients, who survive the acute phase of the disorder, will suffer from PICS under the suppression of T cell autophagy often caused by nutritional stress, insulin infusion, and sustained inflammation, etc.

Autophagy is required to maintain cytokine production. Production of IFN-γ and IL-2 in T cells is decreased due to blockade of Atg7 gene, which is important for macroautophagy (22). Furthermore, in the septic murine model, cytokine production of Th1, Th2, and Th17 were reduced (16). However, we demonstrated that IFN -γ and IL-10 concentrations were elevated in CD4-Cre/Atg5^f/f^ mice. In particular, IL-10 production by T cells from CLP-operated CD4-Cre/Atg5^f/f^ mice was markedly higher than that of the control mice, suggesting autophagy has a role in controlling cytokine production to some extent. The plausibility of this result is supported by the report that macrophages without the autophagy protein Atg16L1 show enhanced IL-1β and IL-18 production by LPS stimulation (23). Furthermore, inhibition of autophagy promotes IL-1β production in macrophages and dendritic cells, and augments secretion of IFN-γ by T cells (24). Consequently, sustained IL-10 production likely contributes to compensatory anti-inflammatory response syndrome and leads to immunosuppression together with apoptosis of immune cells. Supporting the above explanation, we observed a significant difference in mortality between the CD4-Cre/Atg5^f/f^ mice and the control mice using the less severe CLP model. However, the survival benefit was more robust in control mice at around 72 hours after CLP surgery, which is thought to have been caused by the beneficial effects of Atg5 on T cell viability.

We have demonstrated that blocking autophagy leads to apoptosis and immunosuppression in concordance with an insufficient autophagy process in T cells. To summarize, acceleration of autophagy might alleviate immunosuppression. We can speculate on several treatment candidates that modulate autophagy kinetics for immunosuppression. The efficacy of these interventions for T cell autophagy should be clarified in the future investigations.

## CONCLUSIONS

We demonstrated that blocking autophagy accelerated apoptosis and increased mortality in concordance with the insufficient autophagy process in CD4^+^ T cell in the septic murine model, suggesting that T cell autophagy plays a protective role against apoptosis and immunosuppression in sepsis.

## ACKNOWLEDGMENTS

We thank Dr. Noboru Mizushima (University of Tokyo, Japan) for kindly gifted green fluorescent protein (GFP)-microtubule-associated protein light chain 3 (LC3) transgenic mice and Atg5^f/f^ mice; Dr. Masafumi Arima (institute of Dokkyou medical university, Japan) for kindly gifted CD4 T cell Cre recombinase (CD4-Cre) mice; Mrs. Aya Goda and Yoichi Teratake for their excellent technical assistance; Dr. Paul E. Swanson (Foothills Medical Centre, Canada) for validation of pathologic findings; and Ms. Yoshiko Ohashi for her excellent secretarial assistance.

## Supplementary Material
