# Supplementary figures and images for: Suppression of T Cell Autophagy Results in Decreased Viability and Function of T Cells Through Accelerated Apoptosis in a Murine Sepsis Model*
Source: Crit Care Med. 2016 Dec 16;45(1):e77–85. doi: 10.1097/CCM.0000000000002016 (PMC5364514; doi:10.1097/CCM.0000000000002016)

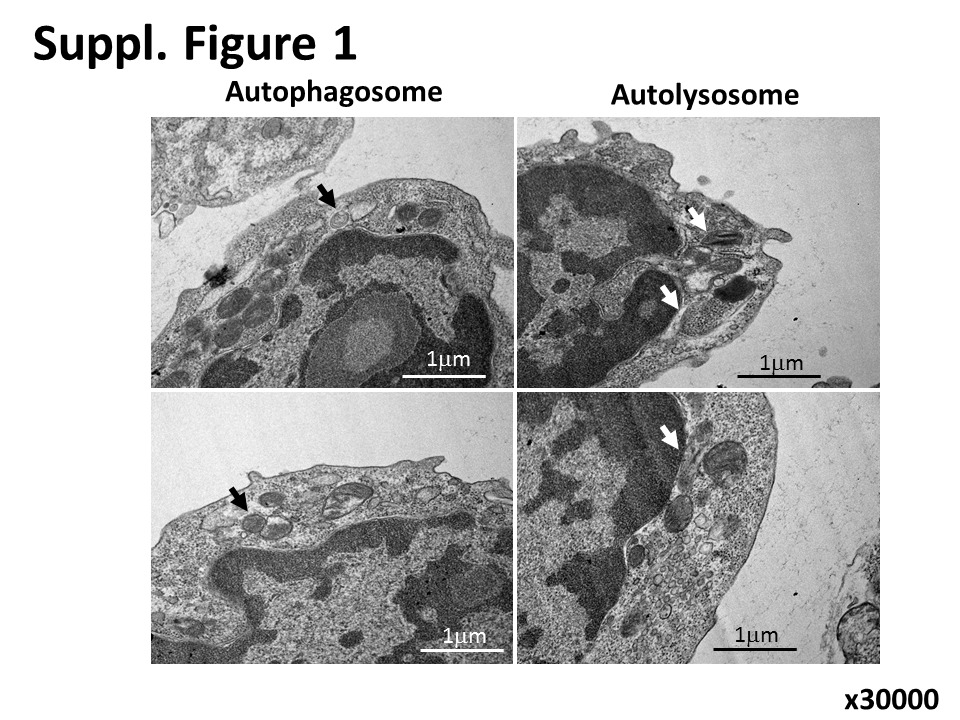

Supplement: Supplementary file 1 [file ccm-45-e77-s001.tif]

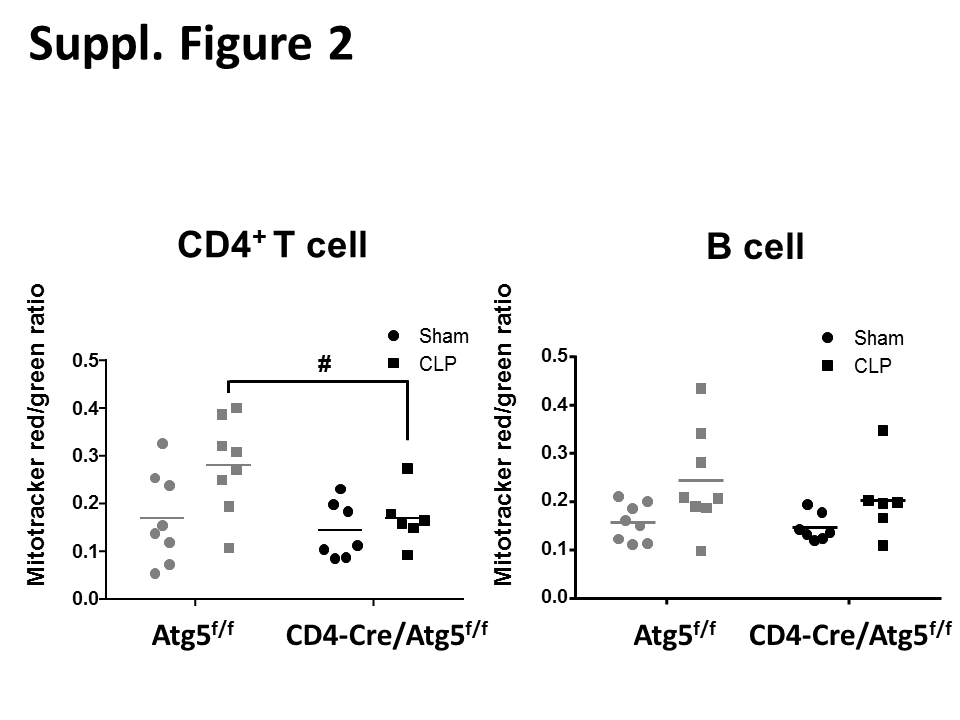

Supplement: Supplementary file 2 [file ccm-45-e77-s002.tif]

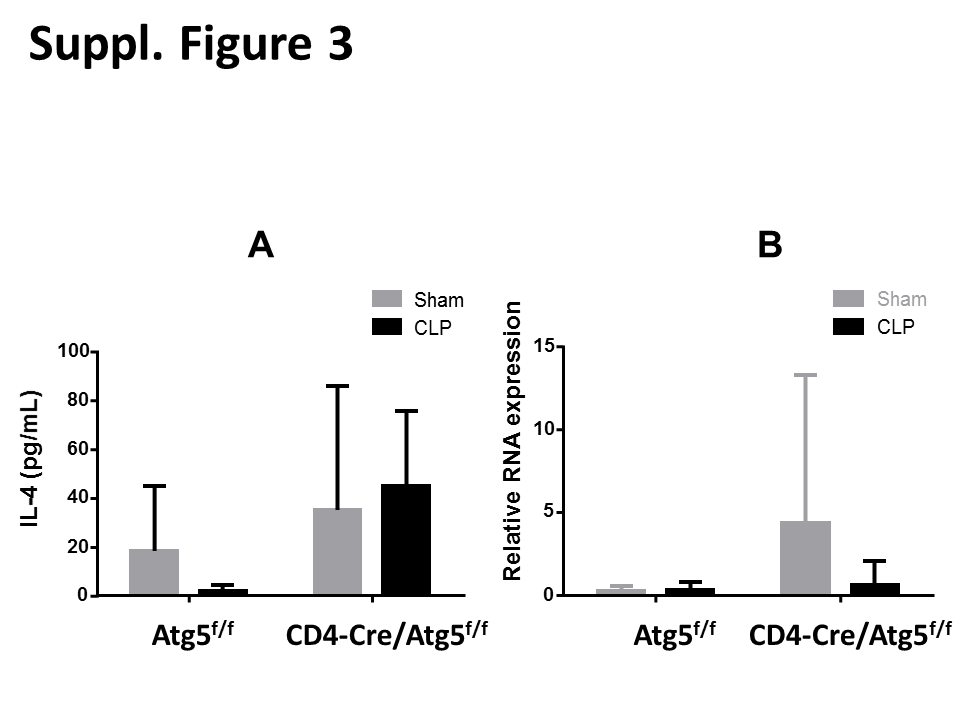

Supplement: Supplementary file 3 [file ccm-45-e77-s003.tif]
